# Semi-Supervised Nests of Melanocytes Segmentation Method Using Convolutional Autoencoders

**DOI:** 10.3390/s20061546

**Published:** 2020-03-11

**Authors:** Dariusz Kucharski, Pawel Kleczek, Joanna Jaworek-Korjakowska, Grzegorz Dyduch, Marek Gorgon

**Affiliations:** 1 Department of Automatic Control and Robotics, AGH University of Science and Technology, al. A. Mickiewicza 30, 30-059 Krakow, Poland; pkleczek@agh.edu.pl (P.K.); jaworek@agh.edu.pl (J.J.-K.); mago@agh.edu.pl (M.G.); 2 Chair of Pathomorphology, Jagiellonian University Medical College, ul. Grzegorzecka 16, 31-531 Krakow, Poland

**Keywords:** deep learning, autoencoders, semi-supervised learning, computer vision, pathology, epidermis, skin

## Abstract

In this research, we present a semi-supervised segmentation solution using convolutional autoencoders to solve the problem of segmentation tasks having a small number of ground-truth images. We evaluate the proposed deep network architecture for the detection of nests of nevus cells in histopathological images of skin specimens is an important step in dermatopathology. The diagnostic criteria based on the degree of uniformity and symmetry of border irregularities are particularly vital in dermatopathology, in order to distinguish between benign and malignant skin lesions. However, to the best of our knowledge, it is the first described method to segment the nests region. The novelty of our approach is not only the area of research, but, furthermore, we address a problem with a small ground-truth dataset. We propose an effective computer-vision based deep learning tool that can perform the nests segmentation based on an autoencoder architecture with two learning steps. Experimental results verified the effectiveness of the proposed approach and its ability to segment nests areas with Dice similarity coefficient 0.81, sensitivity 0.76, and specificity 0.94, which is a state-of-the-art result.

## 1. Introduction

Over the past few decades, both incidence and mortality rates caused by cutaneous melanoma (the most aggressive and lethal skin cancer) among Caucasian populations worldwide has significantly increased [1]. In many countries and regions of the world (such as France, Australia, and Switzerland), the crude incidence rate of cutaneous melanoma increased by 50%–100% between 1993 and 2013 [2]. According to surveys conducted by national health services, melanoma is currently responsible for nearly 70% of all skin cancer-related deaths in the United States and in Australia [3,4]. Although, to date, no effective treatment of melanoma in advanced stages has been developed, an early melanoma (in the in situ stage) is treatable in about 99% of the cases with all but a simple excision [5]. Therefore, the early diagnosis of melanoma, and especially distinguishing melanoma from other types of skin melanocytic lesions, has become an extremely important issue.

The gold standard for diagnosing skin melanocytic lesions (including melanoma) is the histopathological examination—the microscopic examination of tissue in order to study the manifestations of disease [6]. The histopathological criteria for diagnosing skin melanocytic lesions are based on the analysis of such features as lesion’s asymmetry, morphometric features of epidermis, proliferation patterns of single melanocytes, and more [7,8]. In particular, the size, shape, position, and distribution of nests of melanocytes (i.e., aggregations of melanin-producing cells, located originally in the bottom layer of the epidermis) are considered. The traditional histopathological examination was carried out manually and with the use of a light microscope. However, such an approach has at least two important drawbacks: quantitative analysis of a large volume of specimens is a laborious activity, and it is a subjectivity-prone task whose results are not reproducible [9,10]. Therefore, there is a need to develop automatic image analysis methods for the diagnostics of skin melanocytic lesions, which will provide accurate, reliable, and reproducible results. The rapid development in the fields of digital pathology and artificial intelligence facilitates research works in this topic.

Artificial intelligence research (AI) is a wide-ranging branch of computer science that was already introduced more than half a century ago [11]. However, the greatest progress has been visible in recent years. Deep learning models, which are the latest but also the most promising methods, have been exploited with impressive results in signal processing and computer-vision tasks. Deep learning is currently one of the most popular advanced neural network models used for image segmentation, classification, and reconstruction for challenges that have not been solved yet, particularly in the field of biological systems and medical diagnostics [12]. Progress in the hardware, software, algorithms, and availability of huge datasets allow employment of high accuracy image recognition systems. A systematic review on the use of deep neural networks including convolutional autoencoders can be found in [13,14].

In this paper, we present a new approach that is one of the first approaches to the segmentation of nests of melanocytes. We examine the possibility to use convolutional autoencoders, which are a specific type of feedforward neural networks where the input is the same as the output. They compress the input into a lower-dimensional code and then reconstruct the output from this representation. We firstly train the network in an unsupervised way to obtain the weights for the encoding part; secondly, we train the network with limited ground-truth images to receive the weights for the decoding part. The network architecture is adapted to the specification of our problem including the densely-connected classifier layer.

To the best of our knowledge, it is the first method to segment nests of melanocytes in histopathological images of H&E-stained skin specimens.

The novelty of this work can be summarized as:

- we present a deep learning based solution for the segmentation of nests on histopathological images,
- we propose a convolutional autoencoder neural network architecture with two semi-supervised training stages for the encoding and decoding parts,
- our method enables the adaptation of the autoencoders for limited ground-truth data amount based on data augmentation and autoencoders specification.

This paper is organized in four sections as follows: Section 1 presents the skin cancer awareness and covers background information on deep learning methods including autoencoders, related works, motivation of the undertaken research, medical devices, and the problem of melanoma misdiagnosis. Section 2 shows in detail the methodology used in this research to segment the nests regions including data specification and preparation, the autoencoder architecture, layers, training, and classification stages. Section 3 presents the training parameters, and conducts tests and results as well as visualization and interpretation of the layers outputs. Section 4 exposes the conclusions and suggests new lines of research.

### 1.1. Medical Background

Skin melanocytic lesions are neoplasms derived from epidermal melanocytes. The two principal classes of skin melanocytic lesions are “benign” nevi (with no metastatic potential) and “malignant” melanoma (with a metastatic capacity proportional to its thickness).

**Figure 1:**
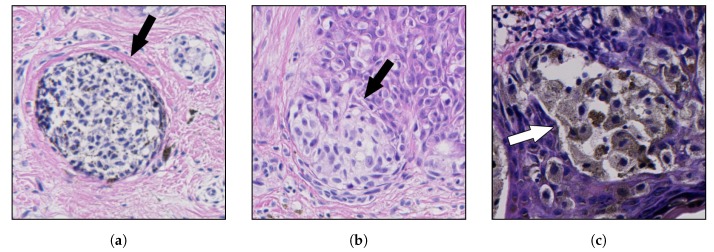
Examples of nests of nevus cells (marked with arrows): (**a**) a dermal nest; (**b**) a nest at the tip of a rete ridge; and (**c**) a nest adjacent to the epidermal plate; note a strong pigmentation of cytoplasm in nevus cells. The structure of nests is highly not-uniform and varies between individual nests.

In the context of melanocytes, the term “nevus” denotes the localized aggregation of nevus cells, i.e., cells derived from melanocytes, as benign neuroectodermal proliferations, neoplasms, or both [15]. Nevus cells, arising as a result of proliferation of melanocytes at the dermal-epidermal junction, are larger than typical melanocytes, do not have dendrites, have more abundant cytoplasm with coarse granules, and are typically grouped in nests [16,17]. Cells within nests are often oval or cuboidal in shape, with clear cytoplasm and variable pigmentation [18]. In the superficial dermis, the cells have an epithelioid cell topography and contain amphophilic cytoplasm with granular melanin. The nuclei have uniform chromatin with a slightly clumped texture. Deeper in the dermis, there is a diminished content of cytoplasm, and the cells resemble lymphocytes and are arranged in linear cords [19]. Examples of nests of nevus cells are shown in Figure 1.

Melanoma is the generic term for all malignant neoplasms derived from melanocytes, with most melanomas developing through an out-of-control progressive proliferation of melanocytes within the squamous epithelium [8]. Melanomas are the most aggressive and dangerous neoplasms; they grow fast and quickly metastasize to nearby lymph nodes and other organs.

In recent years, the sharp dichotomy between “benign” nevi and “malignant” melanoma started to be considered an oversimplification [7]. No histopathological criterion is entirely specific for nevi (all can be occasionally found in a melanoma) [20] and some criteria are infrequent, or have low specificity, or suffer from high inter-observer variation [21].

Examples of criteria which provide strong evidence in favor of melanoma, but are extremely infrequent, include presence of necroses (sensitivity of 15.3%, specificity of 100%) and melanin in deep cells (sensitivity of 36.1%, specificity of 90.4%). On the other hand, some highly sensitive and specific criteria, such as the presence of mitoses (sensitivity of 81.9%, specificity of 100.0%) might be hard to detect automatically, as demonstrated in the course of the MITOS-ATYPIA-14 challenge and Tumor Proliferation Assessment Challenge 2016—the $F_{1}$-score of top-performing methods for mitosis detection was roughly 0.650 [22,23]. The analysis of cases in [21] showed that, in melanomas, except for cytological atypia, not even one of the investigated features was constant.

Moreover, some challenging lesions contain conflicting criteria suggesting opposite diagnoses. Consequently, in many instances, the diagnosis may be subjective because of differences in training and philosophy, and the experience of the observers: accordingly, the same lesion may be classified as dysplastic nevus with severe atypia by one pathologist and as melanoma in situ by another. Therefore, criteria must be used in clusters and ought to be used for specific differential diagnosis (i.e., a specific form of nevus should be differentiated from a type of melanoma morphologically mimicking it) [7]. The above-mentioned issues expose the need for CAD systems which will take into consideration as wide a spectrum of histopathological features as possible.

The histological criteria currently used in the diagnosis of skin melanocytic lesions consist of the analysis of numerous features, such as: lesion’s asymmetry, morphometric features of epidermis, proliferation patterns of single melanocytes and nests of nevus cells, cytological atypia, mitoses, and necroses [7,8]. For nests of nevus cells, the following features are typically considered: size, shape, horizontal and vertical proliferation patterns (as well as the regularity for each of those features). These are nests-related diagnostic criteria for some of the most common types of skin melanocytic lesions:

- In common nevi, the nests are roundish or regularly elongated, and typically positioned at the tips of rete ridges [24].
- Melanocytic nevi contain intraepidermal or dermal collections of nevus cells or both. The cells within the junctional nests have round, ovoid, or fusiform shapes and are arranged in cohesive nests [18].
- One of the major histological criteria for the diagnosis of lentigo maligna (a precursor to lentigo maligna melanoma, a potentially serious form of skin cancer) is the absence of intraepidermal nesting [8].
- A junctional dysplastic nevus consists of a proliferation of a variable combination of single melanocytes and nevus cells in nests along the dermal-epidermal junction. If present, nests are often irregular in size and shape and may “bridge” or join together [24].
- In superficial spreading melanoma (SSM) and melanoma in the in situ stage (i.e., entirely restricted to the epidermis, the dermal-epidermal junction, and epithelial appendages), large, irregularly shaped, confluent nests are unevenly distributed along the dermal-epidermal junction, separated one from another by “skip areas” which are either free of melanocytes or with a lesser number of melanocytes arranged in a lentiginous pattern [7,8]. In SSM, nests are also present above the suprapapillary plates at the edges of rete ridges and a discohesive appearance in large nests is often evident. The cellularity, the pigment, and the type of melanocytes vary greatly among nests.
- Melanomas are subdivided in “radial” and “vertical” growth phases: “radial growth phase” includes melanoma in situ and early invasive superficial spreading melanoma and is characterized by small nests in the papillary dermis, whereas nests larger than those at the junction are typical of the “vertical growth phase” [7].

**Figure 2:**
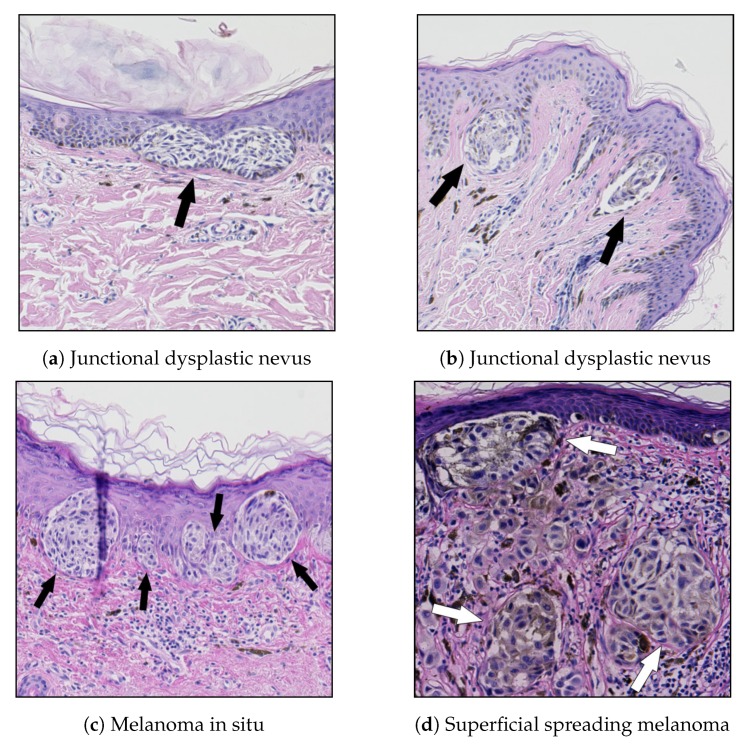
Examples of melanocytic lesions containing nests of nevus cells: (**a**) in junctional dysplastic nevi, nevus cells are typically arranged in cohesive nests along the dermal-epidermal junction nests and often join together; (**b**) in nevi, nests are often positioned at the tips of rete ridges; (**c**) in melanoma in situ, there are often large, confluent nests, irregular in shape and size, unevenly distributed along the dermal-epidermal junction; and (**d**) in SSM, nests are present above the suprapapillary plate.

Examples of the above-mentioned melanocytic skin lesions are shown in Figure 2.

### 1.2. Melanoma Misdiagnosis Problem

The histopathological examination constitutes the gold standard for diagnosing skin melanocytic lesions—other existing forms of examination (such as dermatoscopy) do not yield comparably high diagnostic confidence [25,26]. Nonetheless, even this “gold standard” is far from being perfect and pathologists evaluating the same lesion may not be concordant one with another regarding the diagnosis. Numerous surveys revealed that, even in the case of the histopathological examination, the melanoma misdiagnosis rate may well reach up to 25% [9,27,28,29]. A diagnostic error with particularly severe consequences is a false-negative diagnosis of melanoma (i.e., a situation when a malignant lesion is misdiagnosed as a benign one)—it delays the start of the treatment, which typically leads to further medical complications resulting even in a patient’s death. Three important reasons for the above-mentioned lack of concordance between pathologists and the high melanoma misdiagnosis rate are that histopathological criteria are vaguely defined, in many cases, lesions are evaluated not by dermatopathologists or surgical pathologists but by general histopathologists (who lack profound knowledge of the diagnostic niceties), and the diagnosis is highly subjective as it is based mainly on experience and intuition of a given pathologist.

These obstacles encourage researchers to try to develop new methods for the automatic analysis of histopathological features of skin, such as features related to the nests of melanocytes, which could increase the specificity and sensitivity of the assessment of skin melanocytic lesions.

### 1.3. Image Acquisition

**Figure 3:**
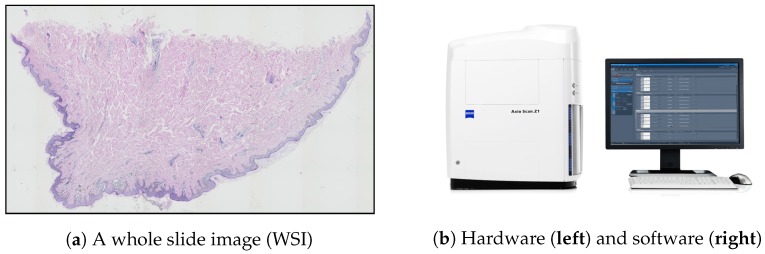
Whole slide imaging: (**a**) an example of a WSI produced by a scanning system; (**b**) a WSI scanning system consists of a dedicated hardware and software.

The problems plaguing the traditional histopathological examination (mentioned in Section 1) may be addressed by digital pathology—a rapidly growing field primarily driven by developments in technology, which is mainly about analyzing whole slide images (WSIs). WSIs are glass slides of tissue specimens digitized at high magnification and thus able to provide global information for quantitative and qualitative image analysis (Figure 3a) [30,31].

To obtain a WSI image, either an automatic microscope or a WSI scanner is typically used. Modern microscopes have a wide variety of components that can be automated by means of electronic control (mainly shutters, stages, light sources, and focus control) and all these motorized components, sensors, and input devices are typically integrated into a software environment. However, the “traditional” microscope systems still have at least one serious limitation in the context of digital pathology: due to the open construction, they do not provide consistent imaging conditions. This shortcoming was eliminated with the introduction of whole slide scanners—a specially designed microscope under robotic and computer control, which has all components assembled in a special casing (Figure 3b) [32,33].

After digital data are captured via the camera’s charge-coupled device (CCD), the virtual slide is assembled together from large numbers of image frames in one of the following ways, depending on the particular scanner being used: tiling, line scanning, dual sensor scanning, dynamic focusing, or array scanning (the process is performed automatically by a specialized imaging software). However, the stitching process rarely produces a truly seamless image, and thus artifacts (e.g., related to vignetting) are typically observed even in images captured using a whole slide scanner.

For most diagnostic work, the digital slides are routinely scanned at ×20 magnification (at resolution approximately 0.25μm/px–0.5μm/px) [34,35]. The size of a high-resolution whole slide image may well be up to 75000 ×50000 px. When stored in an uncompressed format or using lossless compression, such high-resolution digital slides may result in very large files (on the order of several GB), impacting storage costs and work throughput. Consequently, for routine examination by pathologists, a lossy compression technique (e.g., the JPEG or JPEG 2000 image standard) is typically applied. However, since compressing images in a lossy way renders them virtually useless for various methods of automatic digital image processing and analysis [36], for this sort of research, the TIFF format is usually employed to archive virtual slides.

Nonetheless, in recent years, some context aware image compression methods (i.e., techniques, which compress only irrelevant parts of the image, while leaving regions valuable from the diagnostic point of view intact) were proposed—some notable examples are briefly discussed below. Tellez et al. [37] proposed a method to reduce the size of a gigapixel image while retaining semantic information by shrinking its spatial dimensions and growing along the feature direction—an image is firstly divided into a set of high-resolution patches, then each high-resolution patch is compressed with a neural network mapping every image into a low-dimensional embedding vector, and finally each embedding is placed into an array that keeps the original spatial arrangement intact so that neighbor embeddings in the array represent neighbor patches in the original image. Hernández-Cabronero et al. [38] proposed an optimization method called mosaic optimization for designing irreversible and reversible color transforms simultaneously optimized for any given WSI and the subsequent compression algorithm—the method is designed to enable continuous operation of a WSI scanner. Niazi et al. [39] proposed a pathological image compression framework to address the needs of Big Data image analysis in digital pathology, specifically for breast cancer diagnosis, based on a JPEG2000 image compression standard and the JPEG2000 Interactive Protocol—they suggested to identify "hotspots”, i.e., areas in which Ki-67 nuclear protein staining is most prevalent (Ki-67 is an independent breast cancer prognostic marker), and reduce the compression ratio when processing those areas.

### 1.4. Related Works

There are only a few works in the literature which cover automatic processing of histopathological whole slide images of skin specimens stained with hematoxylin and eosin (H&E), the standard stain in histopathology. Some notable examples include an automated algorithm for the diagnostics of melanocytic tumors by Xu et al. [40] (based on the melanocyte detection technique described in [41] and the epidermis segmentation approach described in [42]), a method capable of differentiating squamous cell carcinoma in situ from actinic keratosis by Noroozi and Zakerolhosseini [43] and a method for classifying histopathological skin images of three common skin lesions: basal cell carcinomas, dermal nevi, and seborrheic keratoses by Olsen et al. [44]. The first two methods are based on classic algorithms for image processing and machine learning, whereas the last one uses deep neural networks. None of the above-mentioned methods considers features related to nests of melanocytes, whereas the size, shape, position, and distribution of nests are among important diagnostic criteria when diagnosing skin melanocytic lesions [7,8].

The characteristics of autoencoders have rendered these models useful in various image processing tasks, such as image denoising and image restoration [45,46]. In particular, they have been successfully used in the field of medical imaging and diagnostics (also for super-resolution images) for image denoising [47], detection of cells [48,49], and the analysis of whole tissue structures [50].

Specifically, two methods for automatic cell segmentation use autoencoders for unsupervised cell detection in histopathological slides: Hou et al. [51] proposed a general-purpose method for nuclei segmentation, whereas Song et al. [52] designed a model to segment erythroid and myeloid cells in bone marrow. These methods are designed to detect individual cells or slightly overlapping cells in cases where the maximum possible size of a cell is precisely determined. However, the nests segmentation task is quite different from the above-mentioned tasks as nests are clusters typically composed of dozens or even hundreds of cells and thus no size- or shape-related criteria can be determined. Moreover, the structure of a nest is highly not-uniform and great inter-nest variability is observed, which further complicate matters.

As histopathological image analysis is the gold standard for diagnosing skin melanocytic lesions and grading skin tissue malignancies, the proposed method will provide a relevant input towards automating this procedure.

## 2. Convolutional Autoencoder Segmentation Method for Nests of Melanocytes

### 2.1. Database Specification

To obtain the WSIs, we established scientific cooperation with the University Hospital in Krakow and with the Chair of Pathomorphology of Jagiellonian University Medical College. The dataset included 70. WSIs of selected melanocytic lesions (each image was taken from a separate case): lentigo maligna (22), junctional dysplastic nevus (20), melanoma in situ (13), and superficial spreading melanoma (15). All the histological sections used in the evaluation were prepared from formalin-fixed paraffin-embedded tissue blocks of skin biopsies (each section was about 4 μm thick) stained with H&E using an automated stainer. The original images were captured under 10× magnification (0.44 μm/px) on Axio Scan.Z1 slide scanner and saved into uncompressed TIFF files whose size varied from 3000×1000 to 20,000 × 30,000 pixels. To verify the results, 39. WSIs (10 of lentigo maligna, 10 of junctional dysplastic nevus, 9 of melanoma in situ, and 10 of superficial spreading melanoma) were paired with the ground truth (binary) segmentation masks of nests of nevus cells prepared manually by an experienced dermatopathologist using GIMP image processing program (i.e., in total, we had 39. manually labeled ground truth images).

#### Hematoxylin and Eosin (H&E) Staining

Hematoxylin and eosin stain (H&E stain) is the most commonly used stain for light microscopy in histopathology laboratories due to its comparative simplicity and ability to demonstrate a wide range of both normal and abnormal cell and tissue components [53]. The hematoxylin component binds to basophilic structures, such as DNA of cell nuclei, and colors them blue, whereas the eosin colors cell acidophilic structures, such as cytoplasm and most connective tissue fibers, in varying shades and intensities of pink, orange, and red [53,54]. Consequently, all relevant tissue structures in skin specimen are stained and effectively the whole area of the specimen appears in color.

Since the staining protocol cannot be fully standardized (as for instance the staining quality depends on the quality of the dyes used), the variation in staining is an important factor to be considered when designing automatic image processing methods for digital pathology [55].

### 2.2. Data Preparation

**Figure 4:**
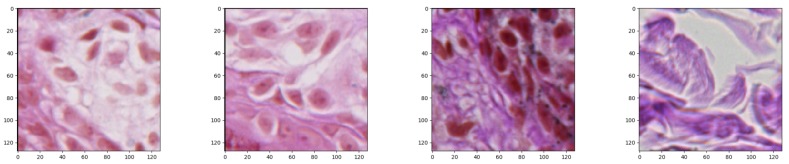
Examples of generated patches of size 128×128 pixels each (windows of such size typically include enough context to label the central pixel as either “part of a nest” or “not part of a nest” with high confidence).

While we were considering a neural network approach to solve the problem of nests segmentation, we were aware of the fact that the input size is strongly related with the final number of parameters that have to be optimized. If the input size is too large, there might be too many model parameters which together with an insufficient number of training examples might lead to overfitting during the training process. For that reason, the input size must be chosen with care. Having a collection of images of different sizes, we decided to split it into small image patches of size 128×128 pixels each, which we found balanced between their size and final model’s accuracy, and set as a neural network input (Figure 4).

Trying to balance classes of the dataset, for those parts of images where nests were not marked, we did not apply an overlapping and simply extracted consecutive examples with 128 px step in width and height. However, while a nest part was found during patches’ extraction, an overlapping was applied in order to produce more examples of particular class. Thanks to that trick and an augmentation technique applied later on, we managed to balance classes in the dataset. The augmentation of the nests images was based mainly on the rotation of particular parts by 45 degrees to generate four more examples. Before the data preparation step was applied, the dataset has been divided into three parts—the training, validation, and test part that have been described in detail in Section 3.1. The dataset split step has been applied at the very beginning of the data preparation process on the images, not patches, in order to provide as reliable results as possible.

### 2.3. The Convolutional Autoencoder

**Figure 5:**
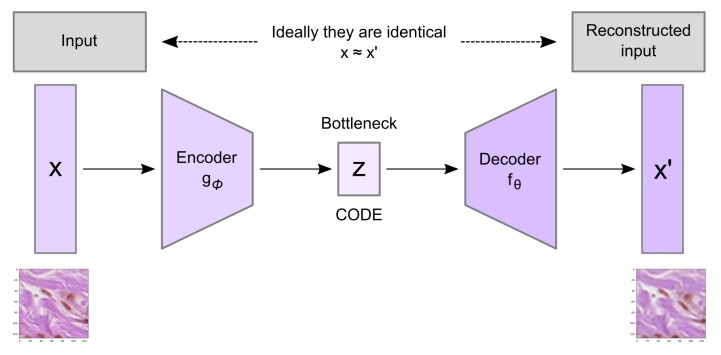
Schema of a basic autoencoder including the encoder, decoder, and code parts. The model contains an encoder function g(.) and a decoder function f(.) parameterized by ϕ and θ, respectively. The low-dimensional code learned for input *x* in the bottleneck layer is *z* and the reconstructed input is $x^{\prime }$.

Convolutional autoencoder architecture imposes a bottleneck in the network which forces a compressed knowledge representation of the original input. An autoencoder consists of three components: the encoder, the code, and the decoder as well as a loss function to compare the output with the target (Figure 5).

Both the encoder and decoder are fully-connected feedforward neural networks where the code is a single layer of a feed forward network (FFN) with specified dimensionality. The encoder function (ϕ) maps the original data *X* to a latent space *F*, which is present at the bottleneck. The decoder function, denoted by θ, maps the latent space *F* at the code to the output, where the output is the same as the input function. Thus, the algorithm is trying to recreate the original image after some generalized nonlinear compression:

(1) $$\begin{array}{c} \phi :X\to F\\ \psi :F\to X\\ \phi ,\psi =\underset{\phi ,\psi }{arg min}\;{\left\|X-(\psi \circ \phi )X\right\|}^{2} \end{array}$$

Autoencoders are trained to minimize the loss function which is a reconstruction error such as mean squared error:

(2) $$L\left(x,x^{\prime }\right)={\left\|x-x^{\prime }\right\|}^{2}={\left\|x-\sigma^{\prime }\left(W^{\prime }\left(\sigma \left(\mathrm{Wx}+b\right)\right)+b^{\prime }\right)\right\|}^{2}$$

where x is usually averaged over some input training set, $W,W^{\prime }$ are weight matrices and $b,b^{\prime }$ are bias vectors for encoder and decoder, respectively.

### 2.4. Convolutional Autoencoder Architecture

**Figure 6:**
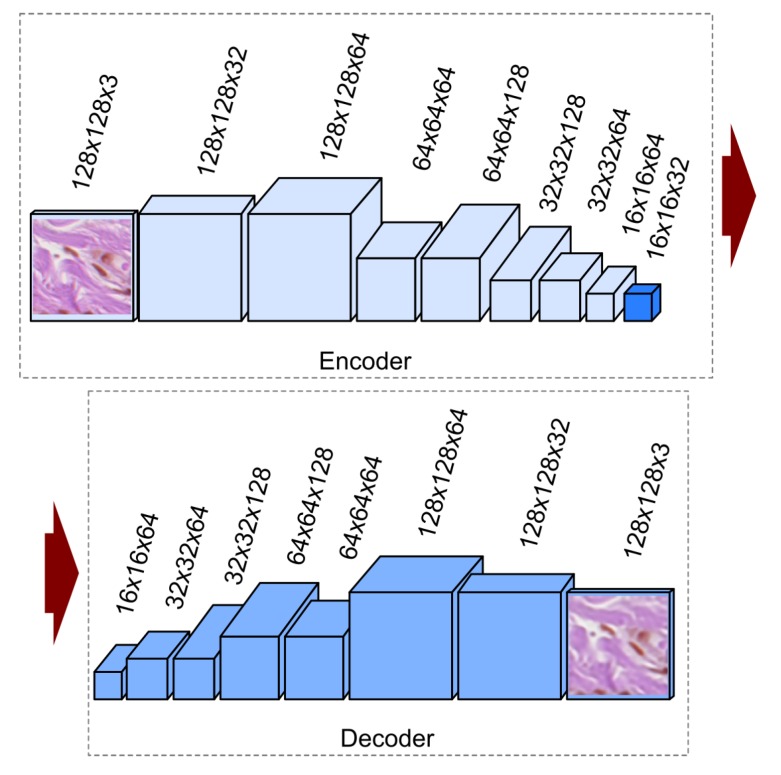
Architecture of the proposed convolutional autoencoder. Each box corresponds to a multichannel feature map. The horizontal arrow denotes transfer between the encoding and decoding parts.

Since our input data consist of images, we use a convolutional autoencoder which is an autoencoder variant where the fully connected layers have been replaced by convolutional layers [56]. The proposed, implemented, and tested convolutional autoencoder architecture has been presented in Figure 6. In our solution, we use the convolution layers with padding along with max-pooling layers in the encoder part. Such combination of layers converts the input image of size 128×128×3 at the RGB colorspace to an image (code) of size 16×16×64 at the latent space. In the decoder part, instead of max-pooling, we use the upsampling layer. The decoder converts the code back to an image at the RGB colorspace in its original size (i.e., 128×128×3).

Convolutional layers apply a convolution operation over the image and perform operation at each point, passing the result to the next layer. Filters belonging to the convolutional layer are trainable feature extractors of size 3×3. As we are solving the segmentation problem, we use padding to avoid spatial dimension decrease. To avoid this, we use ‘same’ padding of size 2 which is correlated with the convolutional layer filter size 3×3 to preserve as much information about the original input volume as possible to extract those low level features which are necessary for the segmentation approach. Each of the convolutional layers stacks is followed by a rectified linear unit (ReLU) activation function. The purpose of this layer is to introduce nonlinearity to our system that basically has just been computing linear operations during the convolutional layers. The ReLU is currently the most popular nonlinear activation function, defined as the positive part of its argument where *x* is the input to a neuron:

(3) f(x)=max(0,x)

Compared to sigmoid function, the ReLU function is computationally efficient, shows better convergence performance, and alleviates the vanishing gradient problem (the issue where the lower layers of the network train very slowly because the gradient decreases exponentially through the layers).

In the encoder part, after the ReLU activation function, we apply a max-pooling layer also referred to as a downsampling layer. The output is the maximum number in every subregion that the 2×2 filter convolves around. The max-pooling layer serves two main purposes: overfitting control and reducing the number of weights (which reduces the computational costs).

The reconstruction process of the autoencoder uses upsampling and convolutions layers. The upsampling layer is a simple layer with no weights, doubling the dimensions of input by repeating the rows and columns of the data. The last convolutional layer is followed by the sigmoid activation function as we are facing the two-class prediction problem while creating the segmentation masks. Sigmoid functions, which are of S-shape, are one of the most widely used activation functions in both machine learning algorithms and deep learning classification layers. A standard choice for a sigmoid function is the logistic function defined as

(4) $$S\left(x\right)=\frac{1}{1+e^{-x}}=\frac{e^{x}}{e^{x}+1}.$$

In Listing 1, we present the proposed autoencoder as well as summarize the layers of the model and their output shapes.

**Listing 1**. Summary of the implemented convolutional autoencoder model for both training stages. In the first training stage, the last convolutional layer has the output shape of (128,128,3) like the input data, while, in the second training stage 128,128,1 as it has been presented in the summary.


Layer (Type)Output ShapeNb. of Param.input_1 (InputLayer)[(None, 128, 128, 3)]0conv2d (Conv2D)(None, 128, 128, 32)896conv2d_1 (Conv2D)(None, 128, 128, 64)18,496max_pooling2d (MaxPooling2D)(None, 64, 64, 64)0conv2d_2 (Conv2D)(None, 64, 64, 64)36,928conv2d_3 (Conv2D)(None, 64, 64, 128)73,856max_pooling2d_1 (MaxPooling2D)(None, 32, 32, 128)0conv2d_4 (Conv2D)(None, 32, 32, 128)147,584conv2d_5 (Conv2D)(None, 32, 32, 64)73,792max_pooling2d_2 (MaxPooling2D)(None, 16, 16, 64)0conv2d_6 (Conv2D)(None, 16, 16, 64)36,928conv2d_7 (Conv2D)(None, 16, 16, 32)18,464conv2d_8 (Conv2D)(None, 16, 16, 32)9248conv2d_9 (Conv2D)(None, 16, 16, 64)18,496up_sampling2d (UpSampling2D)(None, 32, 32, 64)0conv2d_10 (Conv2D)(None, 32, 32, 64)36,928conv2d_11 (Conv2D)(None, 32, 32, 128)73,856up_sampling2d_1 (UpSampling2D)(None, 64, 64, 128)0conv2d_12 (Conv2D)(None, 64, 64, 128)147,584conv2d_13 (Conv2D)(None, 64, 64, 64)73,792up_sampling2d_2 (UpSampling2D)(None, 128, 128, 64)0conv2d_14 (Conv2D)(None, 128, 128, 64)36,928conv2d_15 (Conv2D)(None, 128, 128, 32)18,464conv2d_16 (Conv2D)(None, 128, 128, 1)289
Total params: 822,529

Trainable params: 822,529

Non-trainable params: 0




### 2.5. Semi-Supervised Autoencoder Training Process

**Figure 7:**
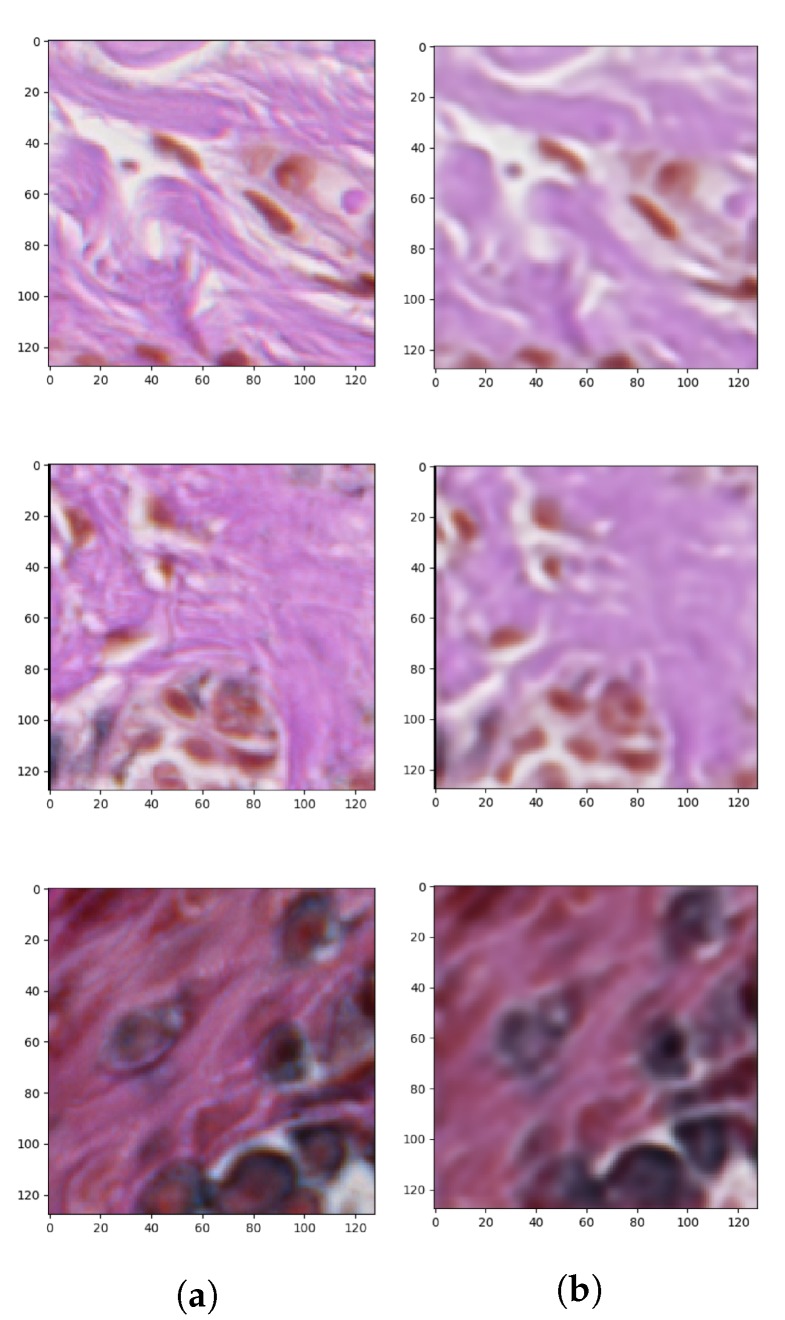
Outcomes of the first stage of autoencoder semi-supervised training process showing: (**a**) original patches and (**b**) reconstructed images.

Since we had only a few ground-truth examples in comparison with the whole dataset of histopathological images, we took advantage of autoencoders with a semi-supervised learning technique to solve the problem of small and insufficient ground-truth dataset. Semi-supervised, in general, consists of three steps. In the first stage, for the unsupervised part, our autoencoder has been trained to reconstruct the input images (Figure 7).

After the first stage of the semi-supervised training process the encoder’s weights have been frozen (fixed) and only the decoder’s weights have been reset in order to train the model again, this time to generate masks. This part is a supervised problem where we use both the input images and segmented outcome mask. In the last step, the fine-tuning has been performed—encoder’s weights have been unlocked again and the whole autoencoder network has been trained again. For the autoencoder training part, the main assumption is that the network can learn to code patterns and structures found in the image in the latent space. The latent size is usually smaller than the input size which enforces the network to choose only those features which best describe the dataset and skip less relevant ones. Later in the second phase, we utilize the feature extraction part—the encoder along with the latent layer—to use those features in order to distinguish (generate masks) structures in the image. Encoder’s weights are blocked because this part of the network is treated as a feature extractor in the second phase of training—while the model for classifying pixels is trained.

During our research, we have analyzed and tested different deep neural network architectures. However, the presented one has been found to be the most effective. The output layer consisted of three filters of size 3×3 with ReLU activation function which produced an output of size 128×128×3 (the same as the input). The mean square error has been chosen as the cost function. We applied learning rate decay to ensure that the cost function’s minimum is not superpassed due to too large weights update as the optimization process continues.

**Figure 8:**
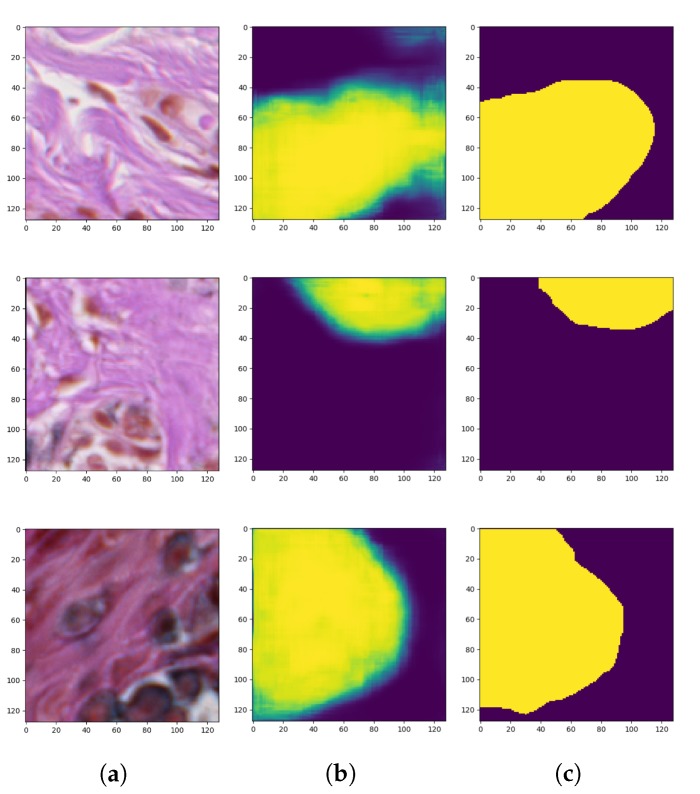
Outcomes of the second training stage of the convolutional autoencoder: (**a**) original images (patches), (**b**) generated masks, and (**c**) ground-truth images.

After we achieved the smallest validation error during the training process of the described autoencoder, the encoder weights were transferred to a new model which had pretty much the same architecture as the one described above. They only differed in the output layer: the autoencoder used for reconstruction produced an RGB image (i.e., an image consisting of three channels), whereas the autoencoder used for segmentation of objects belonging to only one class generates a probability map representing the probability that a given pixel belongs to that class (i.e., an image consisting of only one channel). Therefore, in the autoencoder for the segmentation task which is a binary classification problem, we use the binary cross-entropy loss (BCE) consisting of sigmoid activation function and cross-entropy loss. After applying those changes the training process has been repeated only on supervised examples. This means that the dataset shrunk a lot, but, thanks to initially set weights of the encoder part by the unsupervised training phase, we managed to achieve some decent results. After achieving the minimal training error, in compliance with transfer learning, the pre-trained weights have been unlocked and the fine-tuning has been utilized. Figure 8 presents the generated masks compared to the ground-truth images.

### 2.6. Convolutional Autoencoder Training Parameters

It has been widely observed that hyperparameters are some of the most critical components of any deep architecture. Hyperparameters are variables that determine the network structure and need to be set before training a deep learning algorithm on a dataset. Hyperparameters presented in Table 1 have been chosen experimentally and set before training our autoencoder.

## 3. Experimental Results and Evaluation

In order to evaluate the performance of the semi-supervised training strategy of the autoencoder described in the previous section, we conducted a series of experiments. In this section, we present the training parameters, visualization of the convolutional layers as well as the statistical analysis of the obtained results.

### 3.1. Training, Validation, and Test Sets

The reconstruction stage was performed on all 70. WSIs (both labeled and unlabeled), out of which 49 images (70%) were used for training, 10 (14.3%) for validation, and 11 (15.7%) for testing. The split was performed in a stratification fashion, i.e., in principle, the proportion of the number of labeled to unlabeled images remained constant across all the three sets. The number of patches in each of these sets equaled 223274, 57229, and 116707, respectively.

The segmentation stage was performed on those 39. out of 70 WSIs, which had the ground truth available (i.e., unlabeled images were not used in this stage). Therefore, in this stage, 27 images (69%) were used for training, 6 (15.5%) for validation, and 6 (15.5%) for testing. Each of these sets was created by removing unlabeled images from a corresponding set from the reconstruction stage. The number of patches in training, validation, and test set for the segmentation stage equaled 110736, 31566, and 62718, respectively.

In each stage, the patches were extracted only after splitting the initial dataset (for the given stage) into training, validation, and test sets. Since individual WSIs differed in size, the proportion of patches in those three sets differs between stages.

### 3.2. Visualization of the Convolutional Autoencoders Layers

**Figure 9:**
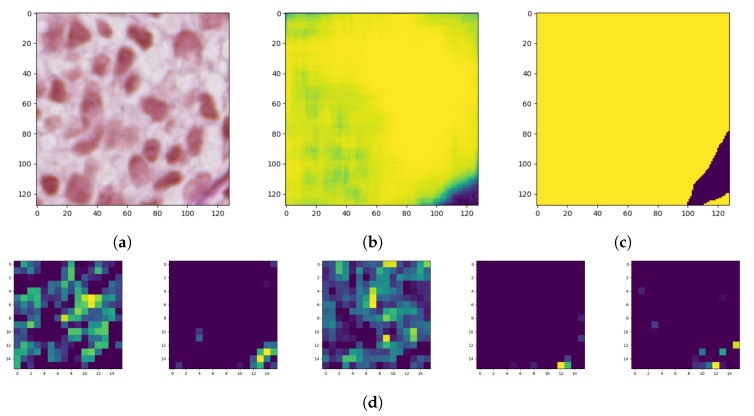
Feature maps of the autoencoder convolutional layers: (**a**) original image, (**b**) generated mask, (**c**) ground-truth image, and (**d**) a partial feature map from latent layers (only a few more interesting activations were included).

Model interpretability of DNN has been an important area of research from the beginning since proposed models achieve high accuracy but at the expense of high abstraction (i.e., accuracy vs. interpretability problem). Visualizing intermediate activations consists of displaying feature maps, filters, and heat maps. The feature maps are the outputs of various convolution and pooling layers in a network. This gives an insight into how an input is decomposed unto the different filters learned by the network (Figure 9). We can observe that the first few layers act as a collection of various edge detectors while the higher-up become increasingly abstract and less visually interpretable.

The reason for visualizing a feature map for a specific input image is to try to gain some understanding of what features are being detected. In the case of classification errors, it can help us to inspect the results and locate specific objects in an image.

During the first step of the training process, we achieved 0.24 mean square error (MSE) on validation set for reconstruction and 0.5 binary cross-entropy for classifying pixels (with threshold equal to 0.5). The within-sample MSE is computed as

(5) $$\mathrm{MSE}=\frac{1}{n}\sum_{i=1}^{n}{\left(Y_{i}-\hat{Y_{i}}\right)}^{2}.$$

where the vector of *n* are predictions generated from a sample of ”n” data points on all variables, *Y* is the vector of observed values of the variable being predicted, with $\hat{Y_{i}}$ being the predicted values.

**Figure 10:**
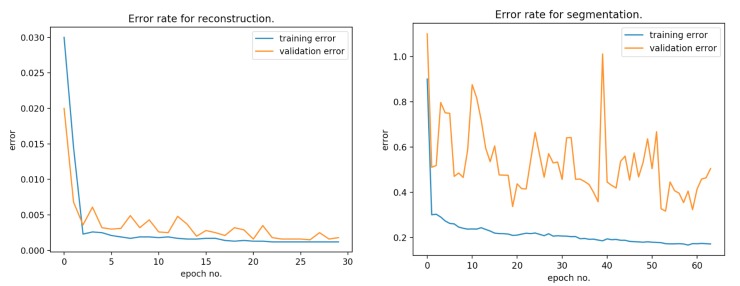
Error rate for reconstruction and segmentation over the training and validation data.

**Figure 11:**
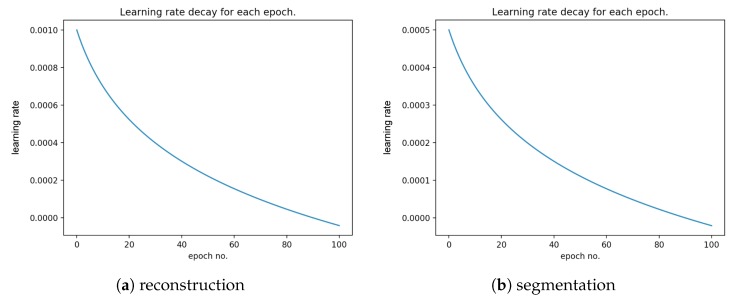
Learning rate decay for each epoch for (**a**) reconstruction and (**b**) segmentation over the training and validation data.

Figure 10 presents the error rate for reconstruction and segmentation over the training and validation data. Figure 11 presents the learning rate decay for each epoch.

One of the most commonly used performance metrics in segmentation problems is the Sørensen index also known as Dice similarity coefficient (DSC), which compares the similarity of two samples from a statistical population [57].

Given two binary sets, *X* and *Y*, the Sørensen’s formula is defined as:

(6) $$\mathrm{DSC}\left(X,Y\right)=\frac{2|X\cap Y|}{\left|X|+|Y\right|}$$

Using the definition of true positive (TP), false positive (FP), and false negative (FN), it can be rewritten as:

(7) $$\mathrm{DSC}=\frac{2\mathrm{TP}}{2\mathrm{TP}+\mathrm{FP}+\mathrm{FN}}$$

where in our case TP denotes correctly detected nests’ structure pixels, FP denotes nests structure pixels not detected, and FP denotes background pixels classified as parts of nests. The DSC is a statistical measure that calculates the degree of overlapping between the experimental segmentation and the manual segmentations where possible values of DSC range from 0.0 to 1.0. A perfect classifier or segmentation model achieves DSC of 1.0.

Furthermore, sensitivity and specificity are calculated using the following equation:

(8) $$\mathrm{SEN}=\frac{\mathrm{TP}}{\mathrm{TP}+\mathrm{FN}}$$

(9) $$\mathrm{SPE}=\frac{TN}{TN+\mathrm{FP}}$$

The proposed algorithm achieved an average DSC of 0.81, sensitivity 0.76, and specificity 0.94. Taking into account that the small ground-truth database has been segmented manually, the achieved DSC score is very promising. We observed that the algorithm’s misclassified areas that were on the border between the patches as well as the area of the nests was really small containing only few cells.

## 4. Conclusions

Our results demonstrate the feasibility of segmenting the nests while using a convolutional autoencoder approaches for a small ground-truth database. The achieved results are very promising as there are no other works or solutions conducted so far. Our method allowed for segmenting the nests with DSC 0.81, sensitivity 0.76, and specificity 0.94, respectively. To the best of our knowledge, this is a first-of-its-kind experiment that shows that convolutional autoencoders may be sufficient for histopathological image analysis with small ground-truth databases. Since no histopathological criterion is entirely specific for nevi (all can be occasionally found in a melanoma) and some challenging lesions contain conflicting criteria suggesting opposite diagnoses, criteria must be used in clusters and ought to be used for specific differential diagnosis—hence, the CAD systems should take into consideration as wide a spectrum of histopathological features as possible. Therefore, the proposed solution for nests segmentation is not intended to be used as a standalone tool for melanoma diagnosis but rather in combination with other automatic diagnostic methods and systems—it might help to increase their accuracy and provide additional grounds for a certain diagnosis, especially for pairs of clinical entities lacking strong delimitation (e.g., junctional dysplastic nevus with severe atypia and melanoma in situ).

### Future Works

Starting from the described framework, as our results seem very promising, there is still much to improve. For example, by cutting out patches from images, we consciously gave up global spatial dependencies in exchange for usable input size. However, spatial information might be preserved by introducing some recurrent structures inside the neural network architecture which would contain some context from previous examples. On the other hand, such a modification in feeding the model with data which can not be random but in a defined, specified order, changes the current approach for data preparation a lot, which in turn will lead to whole algorithm redesign.

In a follow-up study, we intend to improve the architecture of the proposed network as well as train and validate it on a larger dataset containing images from various laboratories. We then plan to integrate the proposed method with our epidermis segmentation method described in [58] and use the information about the distribution of nests of melanocytes within the epidermis to improve the process of diagnosing skin melanocytic lesions (examples of diagnostic information which could be extracted after such a fusion of methods are provided in Section 1.1 Medical Background). Furthermore, high-resolution histopathological images are very large; therefore, image processing, segmentation, and detection are highly compute intensive tasks, and software implementation requires a significant amount of processing time. To assist the pathologists in real time, special hardware accelerators, which can reduce the processing time, are required.

## Figures and Tables

**Table 1:** Parameter settings of the autoencoder.

Parameters	Values
Initializer	Glorot uniform (Xavier uniform)
Number of hidden layers	22
Number of hidden conv. layers	16
Learning rate eq. for reconstruction and segmentation	$\log_{10}\left(0.01\times \mathrm{epoch}+0.1\right)\times 0.001$
Learning rate eq. for fine-tuning	$\log_{10}\left(0.01\times \mathrm{epoch}+0.1\right)\times 0.0005$
Activation function	ReLU and Sigmoid
Batch size	64
Optimizer	Adam
Loss	Mean Squared Error (reconstruction), Binary crossentropy (segmentation)
